# Favorable Marker Alleles for Panicle Exsertion Length in Rice (*Oryza sativa* L.) Mined by Association Mapping and the RSTEP-LRT Method

**DOI:** 10.3389/fpls.2017.02112

**Published:** 2017-12-12

**Authors:** Xiaojing Dang, Bingjie Fang, Xiangong Chen, Dalu Li, Ognigamal Sowadan, Zhiyao Dong, Erbao Liu, Dong She, Guocan Wu, Yinfeng Liang, Delin Hong

**Affiliations:** State Key Laboratory of Crop Genetics and Germplasm Enhancement, Nanjing Agricultural University, Nanjing, China

**Keywords:** chromosome segment substitution line population, favorable marker allele, hybrid seed production, natural population, *Oryza sativa*, panicle exsertion length

## Abstract

The panicle exsertion length (PEL) in rice (*Oryza sativa* L.) is an important trait for hybrid seed production. We investigated the PEL in a chromosome segment substitution line (CSSL) population consisting of 66 lines and a natural population composed of 540 varieties. In the CSSL population, a total of seven QTLs for PEL were detected across two environments. The percentage of phenotypic variance explained (PVE) ranged from 10.22 to 50.18%, and the additive effect ranged from −1.77 to 6.47 cm. Among the seven QTLs, *qPEL10.2* had the largest PVE, 44.05 and 50.18%, with an additive effect of 5.91 and 6.47 cm in 2015 and in 2016, respectively. In the natural population, 13 SSR marker loci were detected that were associated with PEL in all four environments, with the PVE ranging from 1.20 to 6.26%. Among the 13 loci, 7 were novel. The RM5746-170 bp allele had the largest phenotypic effect (5.11 cm), and the typical carrier variety was Qiaobinghuang. An RM5620-RM6100 region harboring the *EUI2* locus on chromosome 10 was detected in both populations. The sequencing results showed that the accessions with a shorter PEL contained the A base, while the accessions with a longer PEL contained the G base at the 1,475 bp location of the *EUI2* gene.

## Introduction

Rice (*Oryza sativa* L.), one of the most important food crops, feeds more than half of the world's population (Sasaki and Burr, 2000). With rapid global population growth and the decreasing availability of arable land, food security has encountered great challenges, and the demand for a high-yielding rice cultivar is a high priority for breeders. Utilization of heterosis is considered one of the most effective strategies for increasing rice yield. To date, hybrid rice technology has been adopted in 27 rice-growing countries in the world (Xie, 2009). In China, hybrid rice has been planted in up to 50% of the total area each year since 1985 (Yuan and Virmani, 1988). The rice hybrid seed production area is ~150,000 hectares annually (Lu and Hong, 1999; Cheng et al., 2004).

**Figure 1:**
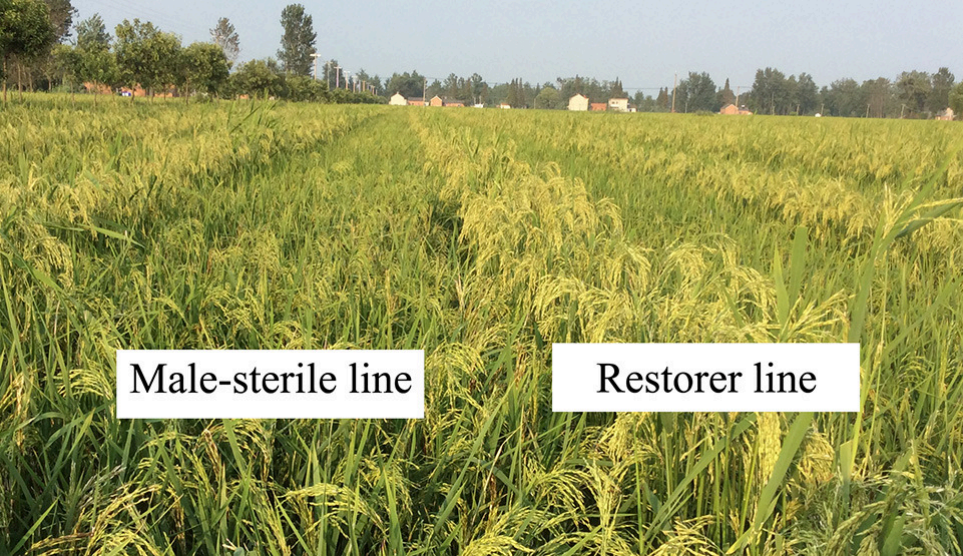
Scene of F_1_ hybrid seed production in the filling stage.

**Figure 2:**
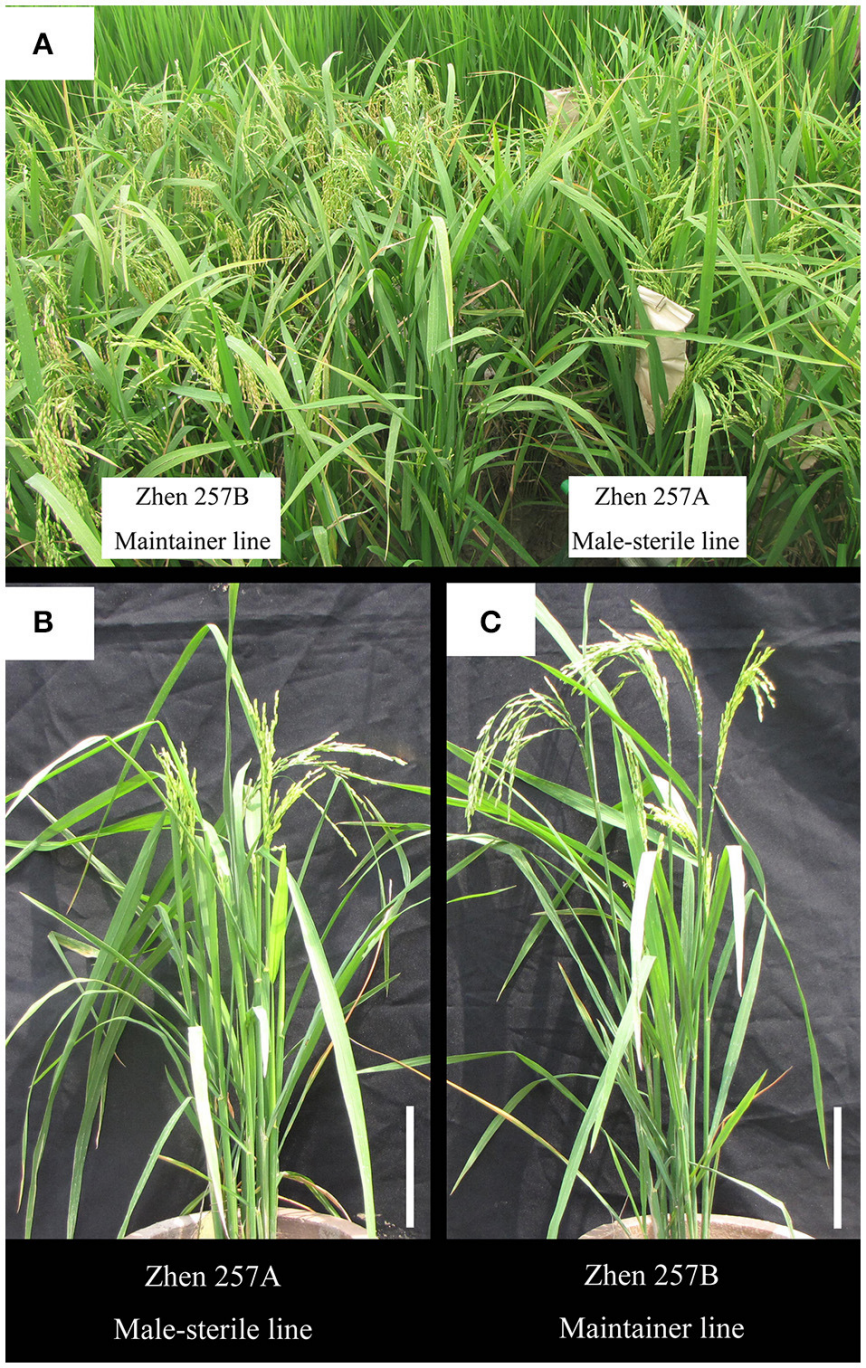
Phenotypes of the maintainer line Zhen 257B, showing no panicle enclosure, and the male-sterile line Zhen 257A, showing panicle enclosure. **(A)** Plants of Zhen 257A and Zhen 257B at the filling stage on the plot. Individual plants of Zhen 257A **(B)** and Zhen 257B **(C)** at the filling stage. Scale bar, 20 cm.

In the process of hybrid rice seed production (Figure 1), cytoplasmic male-sterile (CMS) lines often have a defect of panicle enclosure (Figure 2), which greatly reduces the number of spikelets that can be pollinated by male parents (Shen et al., 1987; Yang et al., 2002). Panicle enclosure in CMS lines is caused by a reduction of the indigenous hormone GA_3_ supply to the panicle, resulting in restricted elongation of the upper internodes. Spraying exogenous gibberellin (GA_3_) on the CMS plants at the initial heading stage in the F_1_ seed production field can eliminate the panicle enclosure. However, spraying a large amount of GA_3_ increases the seed production cost, environmental pollution, and the occurrence of rice kernel smut (Leung et al., 2003; Tsuda et al., 2006; Brooks et al., 2009; Chen et al., 2016). Rutger and Carnahan (1981) found a recessive rice mutant (No. 76:4512) with an upper internode elongation trait derived from the progenies of a japonica rice hybrid and designated it elongated uppermost internode (*eui*) mutant. The mutant shows a dramatically elongated upper internode and is an important germplasm for developing unsheathed-panicle male-sterile lines in hybrid rice breeding. Although the sheathed-panicle in the CMS line is an undesirable phenomenon induced by the sterile cytoplasm, if the panicle exsertion length (PEL) of the maintainer line were increased, panicle enclosure of the CMS line derived from the maintainer line would be alleviated. Therefore, it is important to identify and evaluate more genetic resources and favorable marker alleles of the PEL in different germplasms for hybrid rice seed production.

Following the report by Rutger and Carnahan (1981), several long panicle exsertion germplasms, such as mh-1, Grlc, 02428h, and Xieqingzao eB2, were identified (Wu and Zhang, 1983; Liao et al., 1988; Li et al., 1992; Yang, 1998). Genetic and functional analyses revealed that *EUI1* was located on the long arm of chromosome 5 and encodes a putative cytochrome P450 monooxygenase (Ma et al., 2004, 2006; Luo et al., 2006; Zhu et al., 2006; Magome et al., 2013). In addition to *EUI1*, several other genes related to upmost internode elongation have been cloned and their functions elucidated. *EUI2* encodes a homologous protein of epoxide hydrolase (Zhu, 2003). Four *SUI* (shortened upmost internode) family genes—*SUI1, SUI2, SUI3*, and *SUI4*—encode base-exchange types of phosphatidylserine synthases (Zhu et al., 2011; Yin et al., 2013; Ji et al., 2014). Further, 33 QTLs associated with PEL have been identified using bi-parental segregating populations. These QTL were distributed throughout all 12 chromosomes (Yamamoto et al., 2001; Hittalmani et al., 2003; Qiao et al., 2007; Xiao et al., 2008; Yang et al., 2009; Guan et al., 2011; Ji et al., 2014; Zhao et al., 2015).

QTLs mapped using bi-parental segregating populations reflect the differences of alleles between parents at these loci for the target trait. Association mapping (AM) based on linkage disequilibrium (LD) has been frequently used to search for more than two alleles at a gene locus or additional gene loci and explore the natural genomic diversity at a population level (Zhu et al., 2008; Brachi et al., 2011; Weigel, 2012). AM has been widely applied to exploit favorable marker alleles in rice for many traits, including yield traits (Garris et al., 2005; Agrama et al., 2007; Huang et al., 2010, 2011; Zhao et al., 2011; Li et al., 2012; Vanniarajan et al., 2012; Dang et al., 2015; Yang et al., 2015; McCouch et al., 2016), quality traits (Borba et al., 2010; Huang et al., 2010; Jin et al., 2010; Feng et al., 2016), resistance traits (Famoso et al., 2011; Jia et al., 2012, 2016; Cui et al., 2013, seed vigor traits Cui et al., 2013; Dang et al., 2014; Rebolledo et al., 2015; Phung et al., 2016), and outcrossing traits (Yan et al., 2009; Huang et al., 2010; Dang et al., 2016). Although several studies on associations between markers and the PEL trait in rice have been reported (Begum et al., 2015; Crowell et al., 2016), no significant association has been detected between PEL and molecular markers. It is necessary to enlarge the geographical range of accessions for identifying favorable marker alleles.

The objectives of the present study were (1) to detect QTLs for PEL in a chromosome segment substitution line (CSSL) population using a likelihood ratio test based on stepwise regression (RSTEP-LRT) method; (2) to identify favorable marker alleles of PEL in a natural population composed of 540 accessions collected from 18°N to 54°N by AM; and (3) to compare the relationship between common chromosome regions controlling PEL detected by the two categories of population and the known gene locus.

## Materials and methods

### Plant materials

A CSSL population and a natural population were used. The CSSL population consisted of 66 lines with different A7444 chromosomal segments in the II-32B genetic background (called the IIA-CSSL population hereafter). The total chromosome length of the donor parent A7444 segments in the IIA-CSSL population was ~989.93 Mb (non-idealized CSS lines carrying several substituted segments from the donor parents), covering 94.98% of the complete A7444 genome (She et al., 2017). The natural population was composed of 540 varieties and was the same as that used by Dang et al. (2014). In self-pollinated crop, a natural population is defined as a population that consists of various pure line varieties bred in various places and times within the limits of species. Among the 540 rice accessions, 419 were from China, and 121 were from Vietnam.

### Field planting and trait measurement

For the CSSL population, field experiments were conducted in two environments, i.e., Nanjing (32°07′N, 118°64′E) 2015 (E1) and Nanjing 2016 (E2). For the natural population, field experiments were conducted in four environments, i.e., E1, E2, Yuanyang (35°05′N, 113°97′E) 2015 (E3), and Yuanyang 2016 (E4). The seeds were sown on May 12, and the seedlings were transplanted on June 12 in the E1 and E2 environments. The corresponding dates were May 5 and June 10 in the E3 and E4 environments. The field trials followed a complete randomized block design with two replicates in each environment. Each plot contained 40 plants, with five rows and eight plants in each row. The density was 17 cm between plants, and 20 cm between rows. Standard agronomic management practices were followed.

Twenty to thirty days after the heading dates, five plants in the middle row of each plot were selected to measure the PEL of the main stem panicle. We defined the PEL as follows. If the panicle base node was below the collar of the flag leaf, the PEL was recorded as a negative value; this case is shown in Figure 2B. If the panicle base node was above the collar of the flag leaf, the PEL was recorded as a positive value; this case is shown in Figure 2C. The average value of the five measurements for each accession was used for further analysis.

### DNA extract and PCR amplification

At the tillering stage, a fresh leaf of each accession was collected for DNA extraction. The method of DNA extraction was the same as that described by Gross et al. (2009). The protocols used for SSR amplification, separation of PCR products, and allele recording were the same as those described by Dang et al. (2014).

### Data analysis

All basic statistical analyses were performed in SAS package (SAS Institute Inc., Cary, NC, USA). The calculation method of broad-sense heritability ($H_{B}^{2}$) was the same as that described by Dang et al. (2014).

### QTL detection in the IIA-CSSL population

Since the IIA-CSSL population is not an idealized one, in that each line has a single segment from the donor parent, the standard *t*-test is not suitable for detecting QTLs. Stepwise regression was used to select the most important segments for the trait of interest, and the likelihood ratio test was used to calculate the LOD score of each chromosome segment. This method is statistically equivalent to the standard *t*-test with idealized CSS lines and is designated the RSTEP-LRT (likelihood ratio test based on stepwise regression) mapping method (Wang et al., 2006). The LOD score threshold was arbitrarily set to 3.0, and the QTL detection was completed using the RSTEP-LRT method in the CSL program of QTL IciMapping 4.1 software (Wang et al., 2006). Further, the additive effect and the percentage of phenotypic variance explained (PVE) of each QTL were analyzed. The QTL nomenclature followed the principle reported by McCouch (2008).

### Association analysis in natural population

Rare alleles, i.e., alleles with frequencies of 5% or less, were removed from the dataset before the associations of traits and markers were analyzed. The association analysis of traits and markers was performed with a mixed linear model (MLM) using TASSEL 3.0 (Bradbury et al., 2007). An MLM method can significantly reduce spurious marker-trait associations (type I errors, or false positives) resulting from the population structure because the Q and K matrices are used as covariates. The Q matrix has been reported by Dang et al. (2014), and the K matrix is shown in Table S1. A false discovery rate (FDR) of 0.05 was used as a threshold for significant associations according to the method described by Benjamini and Hochberg (1995). The “null allele” (non-amplified allele, Figure S1) was used to determine the phenotypic effects of other alleles using the identified association locus (Breseghello and Sorrells, 2006).

### Gene sequencing

Sequencing analysis of the allele variation of the *EUI2* gene (Gene ID: *LOC_Os10g35490*) was performed using 27 accessions, and the Nipponbare sequence was used as the reference sequence. Gene-specific PCR primers (forward primer: TCTTTTGCCCCATGCGTTGA; reverse primer: TTCAAACCAAAGCTTCAGCAC) were designed using Primer-BLAST from NCBI (http://www.ncbi.nlm.nih.gov/tools/primer-blast) to amplify the target DNA fragment.

Each 50 μl PCR reaction consisted of 20 mM Tris-HCl (pH 8.0), 100 mM NaCl, 1 mM ethylene diamine tetraacetic acid, 0.1% sodium dodecyl sulfate, 2 nM dNTPs, 0.14 pM forward primers, 0.14 pM reverse primers, 1 U of Super-Fidelity DNA polymerase, and 20 ng of genomic DNA. DNA amplification was performed using a PTC-100^TM^ Peltier Thermal Cycler (MJ Research™ Incorporated, USA) under the following conditions: (1) pre-denaturation at 95°C for 3 min; (2) 35 cycles of denaturation at 95°C for 15 s, annealing at 50°C for 15 s, extension at 72°C for 2 min; and (3) final extension at 72°C for 5 min. The PCR product was gel-purified and sequenced by TsingKe Biological Technology Ltd., Nanjing, China. All sequences were performed using the Sanger double chain termination method by the 3730XL Sequencer of Applied Biosystems Company, USA. Multiple gene and amino acid sequence alignment was performed using the software DNAman 5.2.9.

## Results

### Characteristics of the PEL trait in parents and IIA-CSSL populations

Table 1 shows that the PEL of A7444 (3.2 cm) was significantly longer than that of II-32B (2.0 cm) in both environments. The mean value of the IIA-CSSL population is 2.9 cm, ranging from −3.1 to 12.2 cm. Bidirectional transgressive segregation of the PEL trait was observed in the IIA-CSSL population (Figure S2). The PEL of CSSL64 was −3.3 cm, which was shorter than that of II-32B, and the PEL of CSSL57 was 10.4 cm, which was longer than that of A7444. The results of variance analysis indicated highly significant differences in the PEL trait among the 66 CSSL lines.

**Table 1:** Phenotypic values of the PEL of parents and IIA-CSSLs across two environments.

**Environment**	**Parents**			**IIA-CSSLs**			
	**II-32B mean ± SD**	**A7444 mean ± SD**	***t-*value**	**Mean ± SD**	**Range**	***F* test**	**Heritability in the broad sense %**
E1	2.0 ± 1.2	3.3 ± 1.2	2.7^*^	2.9 ± 3.1	−2.9–12.6	6.7^**^	92.5
E2	2.0 ± 1.2	3.1 ± 0.5	2.6^*^	2.9 ± 3.2	−3.3–11.7	8.9^**^	95.6

*E1 means 2015 in Nanjing; E2 means 2016 in Nanjing*.

* and ** *indicate significance at the 5 and 1% level, respectively*.

### QTL detection in the IIA-CSSL population

Seven QTLs for PEL were detected on chromosomes 1, 8, 10, 11, and 12 in the two environments (Table 2; Figure S3). The PVE ranged from 10.22 to 50.18%, and the additive effect ranged from −1.77 to 6.47 cm. The favorable marker alleles at *qPEL1.1, qPEL8*, and *qPEL10.1* loci were from II-32B, and the favorable marker alleles at *qPEL1.2, qPEL10.2, qPEL11*, and *qPEL12* loci were from A7444. The QTLs *qPEL1.2* and *qPEL10.2* were identified in both E1 and E2. The QTL *qPEL10.2* had the largest PVE, and the PVE was 44.05 and 50.18%, respectively, in E1 and E2. In addition, the additive effect was 5.91 and 6.47 cm in E1 and E2, respectively. The average additive effect over two environments was 6.19 cm, suggesting that the PEL of II-32B would increase 6.19 cm if the two alleles at the *qPEL10.2* locus in II-32B were replaced by the two alleles in A7444.

**Table 2:** QTLs detected for the PEL trait and their genetic parameters in IIA-CSSL population across two environments.

**Locus**	**Chromosome**	**Marker**	**Location/Mb**	**E1**			**E2**		
				**LOD-value**	**Additive effect**	**PVE (%)**	**LOD-value**	**Additive effect**	**PVE (%)**
*qPEL1.1*	1	RM562	14.77	6.72	−3.87	18.92	–	–	–
*qPEL1.2*	1	RM6696	39.98	6.41	3.10	17.84	7.74	4.00	28.37
*qPEL8*	8	RM3572	3.93	–	–	–	4.18	−1.77	13.74
*qPEL10.1*	10	RM5620	17.40	–	–	–	10.53	−3.88	43.00
*qPEL10.2*	10	RM6100	19.35	12.51	5.91	44.05	11.73	6.47	50.18
*qPEL11*	11	RM224	29.52	4.76	2.26	12.44	–	–	–
*qPEL12*	12	RM1246	19.26	4.06	2.15	10.22	–	–	–

*E1 means 2015 in Nanjing; E2 means 2016 in Nanjing*.

### Phenotypic variations in the natural population

**Figure 3:**
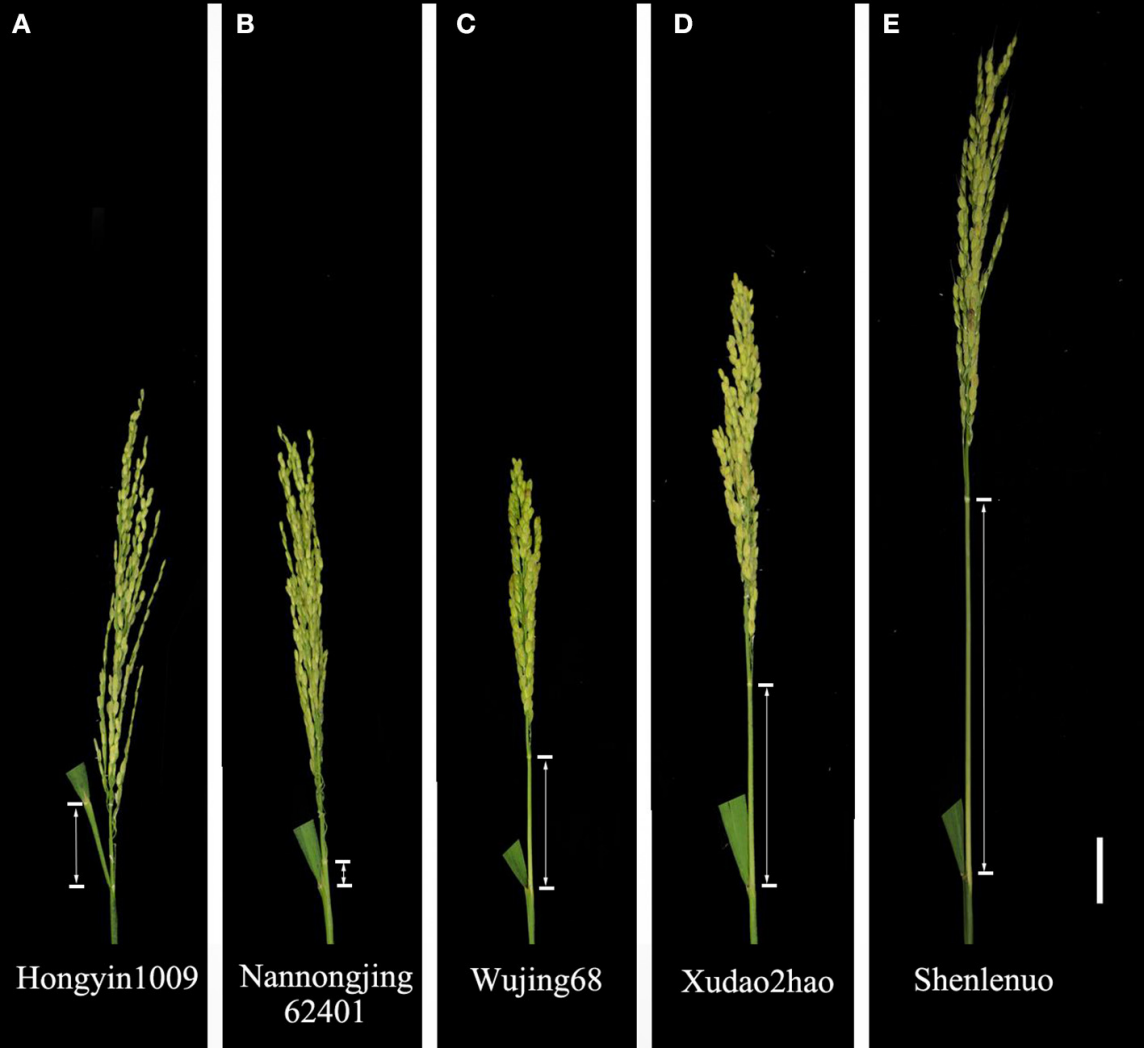
Morphology of the panicle showing the PEL in rice. **(A)** Panicle of accession Hongyin 1009, showing a minimum value of the PEL. **(B)** Panicle of accession Nannongjing 62401. **(C)** Panicle of accession Wujing 68. **(D)** Panicle of accession Xudao 2hao. **(E)** Panicle of accession Shenlenuo, showing a maximum value of the PEL. Scale bar, 3 cm.

The average values over the 540 rice varieties for the PEL were 6.4, 6.1, 6.1, and 6.1 cm for E1, E2, E3, and E4, respectively (Table 3). The rice panicles exhibiting the different values of PEL are shown in Figure 3. The shortest PEL (−5.8 cm) in four environments was found in the variety Hongyin 1009 (Figure 3A). The PELs of Nannongjing 62401 (Figure 3B), Wujing 68 (Figure 3C), and Xudao 2hao (Figure 3D) were 1.4, 4.8, and 10.5 cm, respectively. The largest average value (16.6 cm) of the PEL in four environments was found in the variety Shenlenuo (Figure 3E). A normal distribution for the PEL trait was observed based on the skewness and kurtosis statistics (Table 3, Figure S4). The broad-sense heritability value averaged over four environments was 92.4% for the PEL trait.

**Table 3:** Phenotypic characteristics of the PEL (cm) based on 540 rice accessions in natural population across four environments.

**Environment**	**Mean**	**Standard deviation**	**Maximum**	**Minimum**	**Kurtosis**	**Skewness**	**Heritability in the broad sense %**
E1	6.4	3.9	16.7	−5.8	−0.04	−0.05	93.4
E2	6.1	3.7	16.5	−5.9	0.04	−0.08	92.3
E3	6.1	3.8	16.6	−5.7	0.05	0.04	92.7
E4	6.1	3.7	16.6	−5.8	0.01	−0.06	91.4

*E1 means 2015 in Nanjing; E2 means 2016 in Nanjing; E3 means 2015 in Yuanyang; E4 means 2016 in Yuanyang*.

### SSR loci associated with PEL in the natural population

To reduce the spurious marker-trait associations, we deleted the accessions with Q-value larger than 0.9. Then, the 483 accessions were used to detect the association between the marker and the trait by MLM, in which the Q and K matrices were used as covariates. Thirteen significant association loci were detected in all four environments by MLM under an FDR at the 0.05 level (Table 4). The 13 marker loci were located on chromosomes 1, 2, 3, 5, 6, 8, 9, 10, and 12. The PVE ranged from 1.20 to 6.26%. RM6811 on chromosome 6 had the highest PVE for PEL, explaining phenotypic variance of 5.36% in E1, 4.83% in E2, 6.26% in E3, and 5.69% in E4.

**Table 4:** Marker associated with PEL at *P*-values < 0.05, their equivalent false discovery rate probability (FDR), proportion of PVE, and marker position on chromosome derived from 262 markers and 483 rice accessions.

**Markers**	**Chr**.	**Position/Mb**	**E1**			**E2**			**E3**			**E4**		
			***P*-value**	**PVE/%**	**FDR**	***P*-value**	**PVE/%**	**FDR**	***P*-value**	**PVE/%**	**FDR**	***P*-value**	**PVE/%**	**FDR**
RM283	1	4.89	1.69E-02	3.27	1.84E-02	3.45E-02	2.86	3.61E-02	1.89E-02	3.20	2.94E-02	4.49E-02	2.72	4.50E-02
RM7288	2	9.03	2.28E-02	4.86	2.50E-02	4.70E-02	4.26	5.00E-02	9.94E-03	5.62	1.47E-02	9.22E-03	5.68	1.25E-02
RM6266	3	23.82	1.92E-02	2.44	2.05E-02	2.78E-02	2.24	3.06E-02	4.85E-02	1.93	5.00E-02	3.06E-02	2.15	3.50E-02
RM16	3	23.13	2.22E-02	1.20	2.27E-02	1.24E-02	1.44	1.39E-02	1.52E-02	1.33	2.35E-02	7.00E-03	1.63	1.00E-02
RM159	5	0.49	2.57E-02	4.81	2.73E-02	3.32E-02	4.62	3.38E-02	4.59E-02	4.29	4.71E-02	1.98E-02	4.96	2.25E-02
RM276	6	6.23	3.50E-02	3.11	3.64E-02	3.21E-02	3.16	3.72E-02	3.25E-02	3.20	3.53E-02	3.35E-02	3.20	3.75E-02
RM6811	6	29.23	1.25E-02	5.36	1.36E-02	1.94E-02	4.83	2.22E-02	3.02E-03	6.26	8.82E-03	6.47E-03	5.69	7.50E-03
RM152	8	0.68	3.69E-02	3.01	3.81E-02	4.66E-02	2.86	4.72E-02	1.27E-02	3.67	1.76E-02	9.93E-03	3.79	1.50E-02
RM524	9	17.64	1.38E-03	4.85	2.27E-03	6.56E-04	3.12	2.78E-03	1.03E-03	5.01	2.94E-03	6.49E-04	5.28	2.50E-03
RM410	9	12.92	3.97E-02	2.51	4.09E-02	1.31E-02	3.24	1.67E-02	1.76E-02	3.14	1.98E-02	1.71E-02	3.15	2.00E-02
RM6100	10	17.40	3.78E-03	2.50	6.82E-03	3.00E-03	2.57	5.56E-03	3.20E-03	2.56	3.65E-02	1.53E-02	2.84	1.75E-02
RM269	10	19.35	4.81E-02	2.98	5.00E-02	4.21E-02	3.30	4.44E-02	3.31E-02	3.37	3.82E-02	4.28E-02	3.47	4.70E-02
RM5746	12	5.09	3.67E-03	5.31	4.55E-03	4.19E-03	5.02	8.33E-03	1.50E-03	5.71	5.88E-03	2.11E-03	5.49	5.00E-03

*E1 means 2015 in Nanjing; E2 means 2016 in Nanjing; E3 means 2015 in Yuanyang; E4 means 2016 in Yuanyang*.

### Favorable marker alleles for PEL and their typical carriers

In this study, the marker alleles with positive effects were considered favorable marker alleles for the PEL. The larger the positive effect value, the better the marker allele. A summary of the favorable marker alleles and their typical carrier varieties is shown in Table S2. A total of 26 favorable marker alleles for the PEL were detected across the entire population. The observed allele frequencies for RM16-170 bp and RM6100-145 bp were 82.8 and 83.0%, respectively. The phenotypic effect values of RM524-195 bp, RM159-240 bp, RM269-165 bp, RM152-135 bp, and RM5746-180 bp were more than 3.0 cm on the PEL. The allele RM524-195 bp and RM152-135 bp showed 3.3 and 4.5 cm of the phenotypic effect on the PEL, respectively. In addition, the typical carrier varieties were both Zhongshuyangzhongdao. The phenotypic effect value of RM159-240 bp and RM269-165 bp was 3.6 and 4.2 cm on the PEL, respectively. The typical carrier variety for both of these was Shelenuo. The allele RM5746-180 bp showed the largest phenotypic effect (5.1 cm) on the PEL, and the typical carrier variety was Juhuahuang.

### Differences in DNA sequences at the *EUI2* gene locus between parents of CSSL and among accessions with contrasting PEL

**Figure 4:**
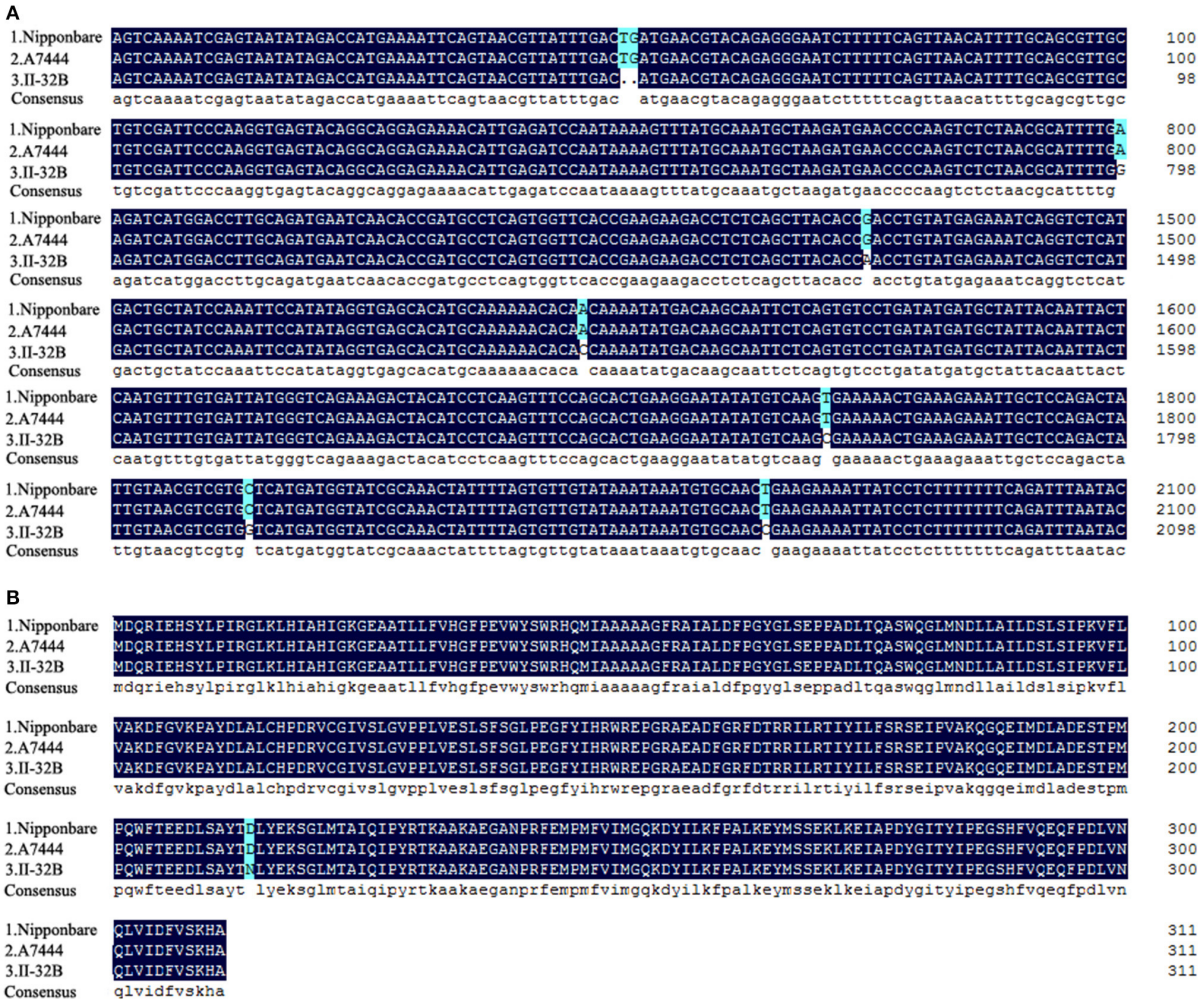
Gene sequence and deduced peptide sequence alignment of *EUI2* between two parents (A7444 and II-32B) and Nipponbare. **(A)** Gene sequence. We show only the alignment results of the variance sequence; the alignment results of the no-difference sequence are not shown. **(B)** Deduced peptide sequence.

In the IIA-CSSL population, we detected a QTL *qPEL10.2* located on the region between RM5620 (17.93 Mb) and RM6100 (19.35 Mb). In the natural population, we detected a region between RM269 (17.40 Mb) and RM6100 (19.35 Mb) on chromosome 10, associated with the PEL trait. The RM5620-RM6100 region is known to harbor a gene *EUI2* locus on chromosome 10. The total length of this gene is 2,114 bp, including five exons. The gene encodes a homologous protein of epoxide hydrolase with 311 amino acids (Zhu et al., 2006). In the IIA-CSSL population, we sequenced DNA of *EUI2* in II-32B and A7444 and aligned it with the reference genome Nipponbare. No difference between A7444 and Nipponbare was found (Figure 4A). Seven variants were found between II-32B and Nipponbare (Figure 4A). The seven variants included six single nucleotide substitutions and one deletion of two bases (Figure 4A). Base A of the fourth exon 1,475 bp in II-32B is replaced by G in A7444, resulting in the transformation of amino acids from asparagine acid to aspartic acid (Figure 4B). The remaining mutations were synonymous. In the natural population, 16 accessions with high values of PEL carrying the RM6100-145 bp allele and nine accessions with low values of PEL carrying the RM6100-135 bp allele were sequenced for the *EUI2* gene. The PEL-values of the 25 accessions are listed in Table S3. After aligning the DNA sequences of *EUI2*, we found that the gene sequences of the accessions with high PEL-values are the same as those of Nipponbare and that the gene sequences of the accessions with low PEL-values show seven variants, in contrast to Nipponbare, which includes six single nucleotide substitutions and one deletion of two bases (Figure S5A). The one deletion of two bases was located in a non-coding region. Among the six single nucleotide substitutions, four were located in a non-coding region and two were located in a coding region. Within the two variants in the coding region, the base A of the accessions with low PEL-values is replaced by G in the accessions with high PEL-values at the location of 1,475 bp, resulting in the transformation of amino acid from asparagine acid to aspartic acid, and the single nucleotide substitutions were synonymous at the location of 1,771 bp (Figure S5B).

## Discussion

In the IIA-CSSL population, we detected seven QTLs for PEL in two environments (Table 2, Figure S3). Among the seven QTLs detected, four QTLs, namely, *qPEL1.2, qPEL10.1, qPEL10.2*, and *qPEL12*, were located on sites near the chromosome location of QTL reported, and three QTLs, *qPEL1.1, qPEL8*, and *qPEL11*, were found to be novel after reviewing previous reports. For the four QTLs reported previously, *qPEL1.2* was located on a site near the *MOC2* gene cloned by Koumoto et al. (2013). The *moc2* mutant showed a low level of cytosolic fructose-1,6-bisphosphatase 1. Defective fructose-1,6-bisphosphatase 1 activity leads to a shortage of sucrose supply, which probably causes the inhibition of tissue elongation. The QTL *qPEL10.1* was located on a site near the *TAW1* gene cloned by Yoshida et al. (2013). *TAW1* encodes a nuclear protein of unknown function and shows high levels of expression in the shoot apical meristem, the inflorescence meristem and the branch meristem. The QTL *qPEL10.2*, which was the main QTL (44.05% PVE in E1 and 50.18% in E2), was located on a site near the *EUI2* gene cloned by Zhu (2003). The QTL *qPEL12*, closely linked with the marker RM1246, was in the same region as that reported by Zhao et al. (2015). For the three new QTLs detected in the present study, the additive effect of QTLs *qPEL1.1* and *qPEL8* was a negative value. The synergistic allelic variations were from II-32B. The PVE of *qPEL1.1* and *qPEL8* was 18.92 and 13.94%, respectively. The *QTL qPEL11* was closely linked with marker RM224 and the favorable marker allele for PEL was RM224-120 bp from parent A7444 (japonica). This favorable marker allele can be used directly for improving the PEL of the japonica rice variety by marker-assisted breeding.

Among the 13 association loci of marker-traits in the natural population identified for the PEL, three marker loci (RM7288 on chromosome 2, RM6811 on chromosome 6, and RM5746 on chromosome 12) exhibited a PVE of more than 5% over four environments (Table 4). Marker locus RM6811 had the largest PVE, with three favorable marker alleles. Allele RM6811-150 bp showed the largest phenotypic effect value (1.7 cm). Fifty of the 483 accessions carried this favorable marker allele, and Qiaobinghuang was the typical carrier variety (possessing the largest phenotypic value among the 50 accessions). Of the two favorable marker alleles found at marker locus RM7288, marker allele RM7288-170 bp had the larger phenotypic effect value (2.14 cm). Forty-seven of the accessions carried this favorable marker allele, and Xiaobaidao was the typical carrier variety. RM5746-170 bp and RM5746-180 bp were the two favorable marker alleles at the RM5746 locus. The former showed the larger phenotypic effect value (5.11 cm), with the typical carrier variety being Qiaobinghuang, and this allele was carried by 36 accessions.

Six of the 13 associations were located on the chromosomal regions where the QTLs (*qPE2, qPE3-2, qPE9, qUIL3b, qEUI10*, and *qUIL10b*) for the PEL had been identified by Yang et al. (2001), Ma et al. (2006), Qiao et al. (2007), Xiao et al. (2008) and Yang et al. (2009) (http://www.gramene.org/). These QTLs are listed in Table S4. The remaining seven association loci (RM283 on chromosome 1, RM159 on chromosome 5, RM276 on chromosome 6, RM6811 on chromosome 6, RM152 on chromosome 8, RM524 on chromosome 9, and RM5746 on chromosome 12) were newly identified in this study. For the seven new loci, 18 favorable marker alleles were identified. Among the 18 alleles, allele RM5746-170 bp showed the largest phenotypic effect value (5.11 cm). Thirty-six of the 483 accessions carried this allele, and Qiaobinghuang was the typical carrier variety (possessing the largest phenotypic value among the 36 accessions).

Marker locus RM6100, which is associated with the PEL, was detected in both the natural population and the IIA-CSSL population. In the IIA-CSSL population, RM6100-145 bp was the favorable marker allele derived from A7444. In the natural population, RM6100-145 bp was also the favorable marker allele. This marker allele was carried by 401 accessions (occupied 83%), and the typical carrier was Shenlenuo. Although the detection principle and method of the two populations for mapping QTLs are different, RM6100-145 bp can be detected consistently, indicating that the allele RM6100-145 bp is conservative.

The *EUI* gene encodes a previously uncharacterized cytochrome P450 monooxygenase, CYP714D1. The monooxygenase catalyzes 16α, 17-epoxidation of non-13-hydroxylated GAs. The 16α, 17-epoxidation reduces the biological activity of GA_4_ in rice. EUI functions as a GA-deactivating enzyme (Zhu et al., 2006). Our results of sequencing and alignment of *EUI* gene showed that a base A of the fourth exon 1475 bp was replaced by G, resulting in the transformation of amino acids from asparagine acid to aspartic acid. We hypothesize that the transformation of amino acids causes deactivation of cytochrome P450 monooxygenase, which hinders 16α,17-epoxidation to non-13-hydroxylated GAs, resulting in accumulation of GA_4_ in upmost internode cells. The accumulation of GA_4_ leads to elongation of the PEL.

Regarding the new loci detected in this study, it is necessary to further narrow the chromosome region and clone the favorable alleles. The mechanism of the PEL genes needs further clarification. It is also necessary to pyramid the favorable alleles into one variety by convergent crossing to breed longer PEL parents for hybrid seed production.

According to the favorable marker allele information, we predict parental combinations to improve the PEL by cross-breeding. The names of the parental combinations and the numbers of favorable alleles that we expect by pyramiding are listed in Table S5. The favorable marker alleles carried by the parents in favorable crosses and the corresponding phenotypic effects are listed in Table S6. Figure S6 shows the morphologies of the panicles of accessions Qiaobinghuang, Zhongshuyangzhongdao, Wanzhongqiu, and Yanglingdao, which are considered superior parents for improving the PEL trait.

## Author contributions

DH: Planned and designed the research; XD, BF, DL, and OS: Performed the field experiment; XD, EL, XC, ZD, DS, GW, and YL: Conducted the molecular experiment; XD: Analyzed the data and wrote the manuscript; and DH: Revised the manuscript. All authors read and approved the manuscript.

### Conflict of interest statement

The authors declare that the research was conducted in the absence of any commercial or financial relationships that could be construed as a potential conflict of interest.

## Supplementary material

Table S1 The K-matrix obtained in this study.

Table S2 Positive favorable marker alleles, phenotypic effect values, and typical varieties for the PEL.

Table S3 The list of the phenotypic value of PEL, of the 25 accessions sequenced.

Table S4 The list of common QTL identified in the natural population of this study and previous studies.

Table S5 Parental combinations and numbers of favorable marker alleles after combinations predicted from AM of the PEL.

Table S6 Favorable marker alleles carried by the superior parents for the PEL and the corresponding phenotypic effect.

Figure S1 Profile amplified by RM6215 using total DNA of partial varieties (Variety A385 to A432) M: Marker, 1-48: rice varieties: 1, Yuedao 114; 2, Yuedao 115; 3, Yuedao 116; 4, Yuedao 117; 5, Yuedao 118; 6, Yuedao 119; 7, Yuedao 120; 8, Yuedao 121; 9, Jia 45; 10, Nannongjing 3786; 11, 24248; 12, Nannongjing 4004; 13, Nannongjing 4016; 14, Zijianwujing; 15, Ningjing 2hao; 16, Wuxiang 99-8; 17, Wuyunjing 8hao; 18, Nannongjing 002; 19, Nannongjing 004; 20, Huaidao 5hao; 21, Zhongzuo 93; 22, Yandao 9hao; 23, Lianjing 4hao; 24, Jindao 1007; 25, Huajing 5hao; 26, Huajing 6hao; 27, Yangfujing 7hao; 28, Yangfujing 8hao; 29, Zhendao 99; 30, Nanjing 42; 31, Lianjing 9823; 32, Huifeng 1hao; 33, Huifeng 2hao; 34, Yandao 8hao; 35, Wuyunjing 21hao; 36, Shashani; 37, Muzhan 4hao; 38, Mudanjiang 29; 39, Mudanjiang 28; 40, Mudanjiang 27; 41, Kenzhan 2hao; 42, Heijing 8hao; 43, Hejing 1hao; 44, Beidao 4hao; 45, Beidao 3hao; 46, Suijing 12hao; 47, Songjing 12; 48, Songjing 11.

Figure S2 Frequency distribution of the PEL in the CSSL population under two growing environments. The parents of II-32B and A7444 were noted above the histograms.

Figure S3 Chromosome locations of the QTLs for the PEL trait detected in the IIA-CSSL population and other reported genes of related traits. The black symbols stand for QTLs detected in E1; the white symbols stand for QTLs detected in E2.

Figure S4 Frequency distribution of the PEL in the natural population under four growing environments.

Figure S5 Gene sequence and deduced peptide sequence alignment of *EUI2* between 25 accessions and Nipponbare. **(A)** Gene sequence. We show only the alignment results of the variance sequence; the alignment results of the no-difference sequence are not shown. **(B)** Deduced peptide sequence.

Figure S6 Morphology of the panicle for four superior parents. Scale bar, 3 cm.
