# Supplementary material for: Favorable Marker Alleles for Panicle Exsertion Length in Rice (Oryza sativa L.) Mined by Association Mapping and the RSTEP-LRT Method
Source: Front Plant Sci. 2017 Dec 12;8:2112. doi: 10.3389/fpls.2017.02112 (PMC5732986; doi:10.3389/fpls.2017.02112)
Supplement: Table S2 — Positive favorable marker alleles, phenotypic effect values, and typical varieties for the PEL. [file Table2.DOC]

**Table S2** Positive favorable marker alleles, observed allele frequency, phenotypic effect value and typical varieties for panicle exsertion length

| Loci-alleles | Observed allele frequency  /% | Phenotypic effect value /cm | | | | | | Typical carrier varieties |
| --- | --- | --- | --- | --- | --- | --- | --- | --- |
|  |  | 2015 | |  | 2016 | | Mean |  |
|  |  | Nanjing/E1 | Yuanyang/E2 |  | Nanjing/E3 | Yuanyang/E4 |  |  |
| RM283-150 | 19.25 | 3.2 | 2.93 |  | 2.57 | 2.44 | 2.79 | Qiaobinghuang |
| RM283-160 | 10.56 | 1.78 | 1.71 |  | 1.83 | 1.72 | 1.76 | Kuobanzhong |
| RM283-165 | 18.63 | 0.63 | 0.75 |  | 0.92 | 1.1 | 0.85 | Wanzhongqiu |
| RM7288-170 | 9.73 | 2.16 | 1.87 |  | 2.19 | 2.32 | 2.14 | Xiaobaidao |
| RM7288-210 | 18.01 | 2.18 | 2.05 |  | 2.1 | 1.91 | 2.06 | Qiaobinghuang |
| RM6266-155 | 13.87 | 3.3 | 3.2 |  | 2.48 | 2.32 | 2.83 | Qiaobinghuang |
| RM16-170 | 82.82 | 1.9 | 1.88 |  | 2.04 | 1.95 | 1.94 | Shenlenuo |
| RM159-240 | 14.49 | 3.34 | 3.53 |  | 3.79 | 3.81 | 3.62 | Shenlenuo |
| RM276-125 | 11.39 | 1.14 | 1.13 |  | 1.25 | 1.49 | 1.25 | Juhuahuang |
| RM276-130 | 20.91 | 2.18 | 2.39 |  | 2.11 | 1.93 | 2.15 | Shenlenuo |
| RM276-135 | 12.22 | 3.04 | 3 |  | 2.93 | 2.82 | 2.95 | Zhongshuyangzhongdao |
| RM6811-140 | 14.70 | 1.16 | 1.22 |  | 1.3 | 1.59 | 1.32 | Zhongshuyangzhongdao |
| RM6811-145 | 11.18 | 1.21 | 1.41 |  | 1.5 | 1.39 | 1.38 | Shenlenuo |
| RM6811-150 | 10.35 | 1.8 | 1.7 |  | 1.61 | 1.75 | 1.72 | Qiaobinghuang |
| RM152-135 | 7.87 | 4.18 | 4.27 |  | 4.81 | 4.84 | 4.53 | Zhongshuyangzhongdao |
| RM152-145 | 32.30 | 1.46 | 1.25 |  | 1.27 | 1.39 | 1.34 | Qiaobinghuang |
| RM152-155 | 8.70 | 2.58 | 2.35 |  | 2.14 | 2.07 | 2.29 | Yanglingdao |
| RM524-170 | 14.29 | 0.61 | 0.75 |  | 0.5 | 0.71 | 0.64 | Xiaobaidao |
| RM524-185 | 21.33 | 1.62 | 1.57 |  | 1.76 | 1.8 | 1.69 | Shenlenuo |
| RM524-195 | 6.83 | 2.98 | 3.05 |  | 3.45 | 3.61 | 3.27 | Zhongshuyangzhongdao |
| RM410-180 | 32.30 | 0.51 | 0.69 |  | 0.73 | 0.89 | 0.71 | Shenlenuo |
| RM410-190 | 20.70 | 0.93 | 0.9 |  | 0.67 | 0.69 | 0.8 | Yanglingdao |
| RM269-165 | 43.89 | 4.31 | 4.12 |  | 4.23 | 4.08 | 4.19 | Shenlenuo |
| RM6100-145 | 83.02 | 2.48 | 2.36 |  | 2.41 | 2.52 | 2.44 | Shenlenuo |
| RM5746-170 | 7.45 | 5.04 | 5.05 |  | 5.12 | 5.23 | 5.11 | Qiaobinghuang |
| RM5746-180 | 9.11 | 2.52 | 2.33 |  | 2.17 | 2.45 | 2.37 | Juhuahuang |
