# Supplementary material for: Favorable Marker Alleles for Panicle Exsertion Length in Rice (Oryza sativa L.) Mined by Association Mapping and the RSTEP-LRT Method
Source: Front Plant Sci. 2017 Dec 12;8:2112. doi: 10.3389/fpls.2017.02112 (PMC5732986; doi:10.3389/fpls.2017.02112)
Supplement: Table S3 — The list of the phenotypic value of PEL, of the 25 accessions sequenced. [file Table3.DOCX]

**Table S3** The list of the phenotypic value of PEL of the 25 accessions sequenced

| Code | Accession name | Phenotypic vale of PEL /cm | Code | Accession name | Phenotypic vale of PEL / cm |
| --- | --- | --- | --- | --- | --- |
| 1 | Wanhuangdao | 13.6 | 17 | Yuedao 21 | -3.5 |
| 2 | Yagnlingdao | 13.9 | 18 | Yuedao 25 | -1.8 |
| 3 | Qaiobinghuang | 13.4 | 19 | Yuedao 26 | -1.2 |
| 4 | Tiejingqing | 13.5 | 20 | Yuedao 33 | -2.9 |
| 5 | Xiaobaiyedao | 13.9 | 21 | Yuedao 34 | -2.5 |
| 6 | Manyedao | 14.3 | 22 | Yuedao 45 | -1.9 |
| 7 | Zhognshuyangzhongdao | 13.7 | 23 | Yuedao 64 | -2.4 |
| 8 | Duiguzhong | 13.5 | 24 | Yuedao 65 | -2.1 |
| 9 | Yilimang | 13.4 | 25 | Heimixiandao | -2.6 |
| 10 | Wuqitou | 13.1 |  |  |  |
| 11 | Jiucaiqing | 13.3 |  |  |  |
| 12 | Juhuahuang | 13.4 |  |  |  |
| 13 | Wanzhognqiu | 13.1 |  |  |  |
| 14 | Xiaobaidao | 14.8 |  |  |  |
| 15 | Shenlenuo | 16.6 |  |  |  |
| 16 | Yuedao 119 | 14.5 |  |  |  |
