# Supplementary material for: Favorable Marker Alleles for Panicle Exsertion Length in Rice (Oryza sativa L.) Mined by Association Mapping and the RSTEP-LRT Method
Source: Front Plant Sci. 2017 Dec 12;8:2112. doi: 10.3389/fpls.2017.02112 (PMC5732986; doi:10.3389/fpls.2017.02112)
Supplement: Table S4 — The list of common QTL identified in the natural population of this study and previous studies. [file Table4.DOC]

**Table S4** The list of common QTL identified in the natural population of this study and previous studies

| SSR  marker | Chromosome | Start position  /bp^a^ | End position  /bp^a^ | QTL reported in the previous studies | | |
| --- | --- | --- | --- | --- | --- | --- |
|  |  |  |  | Start position  /bp^a^ | End position /bp^a^ | Reference |
| RM7288 | 2 | 9,033,547 | 9,033,882 | 8,984,645 | 17,043,505 | Xiao *et al*., (2008) |
| RM6266 | 3 | 23,821,943 | 23,822,102 | 21,544,181 | 24,595,466 | Qiao et al. (2007) |
|  |  |  |  | 22,400,917 | 25,112,877 | Yang et al. (2009) |
| RM16 | 3 | 23,126,064 | 23,126,231 | 21,544,181 | 24,595,466 | Qiao et al. (2007) |
|  |  |  |  | 22,400,917 | 25,112,877 | Yang et al. (2009) |
|  |  |  |  | 8,181,251 | 32,443,016 | Xiao et al. (2008) |
| RM410 | 9 | 17,642,699 | 17,643,295 | 16,271,090 | 18,810,331 | Yang et al. (2009) |
| RM269 | 10 | 17,401,639 | 17,401,724 | 14,270,575 | 17,701,183 | Qiao *et al*., (2007) |
| RM6100 | 10 | 19,351,342 | 19,351,483 | 18,655,588 | 26,086,421 | Yang et al., (2001) |
