# Supplementary material for: Favorable Marker Alleles for Panicle Exsertion Length in Rice (Oryza sativa L.) Mined by Association Mapping and the RSTEP-LRT Method
Source: Front Plant Sci. 2017 Dec 12;8:2112. doi: 10.3389/fpls.2017.02112 (PMC5732986; doi:10.3389/fpls.2017.02112)
Supplement: Table S5 — Parental combinations and numbers of favorable marker alleles after combinations predicted from AM of the PEL. [file Table5.DOC]

**Table S5** Parental combinations and their numbers of favorable marker alleles predicted from the results of association mapping of PEL

| Parental combination | No. of favorable marker alleles predicted | Increments of PEL predicted  /cm |
| --- | --- | --- |
| Zhongshuyangzhongdao× Qiaobinghuang | 13 | 39.39 |
| Zhongshuyangzhongdao× Yanglingdao | 13 | 38.25 |
| Zhongshuyangzhongdao× Wanzhongqiu | 12 | 35.07 |
| Yanglingdao× Shenlenuo | 13 | 32.68 |
| Qiaobinghuang× Wanzhongqiu | 13 | 32.67 |
