# Supplementary material for: Favorable Marker Alleles for Panicle Exsertion Length in Rice (Oryza sativa L.) Mined by Association Mapping and the RSTEP-LRT Method
Source: Front Plant Sci. 2017 Dec 12;8:2112. doi: 10.3389/fpls.2017.02112 (PMC5732986; doi:10.3389/fpls.2017.02112)
Supplement: Table S6 — Favorable marker alleles carried by the superior parents for the PEL and the corresponding phenotypic effect. [file Table6.DOC]

**Table S6** Favorable marker alleles carried by the superior parents for panicle exsertion length and corresponding phenotypic effect

| Superior parent | Locus-allele (Corresponding phenotypic effect value) |
| --- | --- |
| Zhongshuyangzhongdao | RM283-150(2.79), RM16-170(1.94), RM159-240(3.62), RM276-135(2.95), RM6811-140(1.32), RM152-135(4.53), RM524-195(3.27), RM269-165 (4.19), RM6100-145(2.44), RM5746-170(5.11) |
| Qiaobinghuang | RM283-150(2.79), RM7288-210(2.06), RM6266-155(2.83), RM16-170(1.94), RM276-130(2.15), RM6811-150(1.72), RM152-145(1.34), RM524-185 (1.69), RM269-165(4.19), RM6100-145(2.44), RM5746-170(5.11) |
| Shenlenuo | RM16-170(1.94), RM159-240(3.62), RM276-130(2.15), RM6811-145(1.38), RM524-185(1.69), RM410-180(0.71), RM269-165(4.19), RM6100-145(2.44) |
| Yanglingdao | RM283-150(2.79), RM7288-210(2.06), RM6266-155(2.83), RM16-170(1.94), RM276-130(2.15), RM6811-150(1.72), RM152-145(1.34), RM524-185 (1.69), RM410-190(0.80), RM269-165(4.19), RM6100-145(2.44), RM5746-170(5.11) |
| Wanzhongqiu | RM283-165(0.85), RM7288-170(2.14), RM16-170(1.94), RM159-240(3.62), RM276-130(2.15), RM6811-145(1.38), RM152-145(1.34), RM524-170 (0.64), RM410-180(0.71), RM269-165(4.19), RM6100-145(2.44) |
