# Supplementary figures and images for: Favorable Marker Alleles for Panicle Exsertion Length in Rice (Oryza sativa L.) Mined by Association Mapping and the RSTEP-LRT Method
Source: Front Plant Sci. 2017 Dec 12;8:2112. doi: 10.3389/fpls.2017.02112 (PMC5732986; doi:10.3389/fpls.2017.02112)

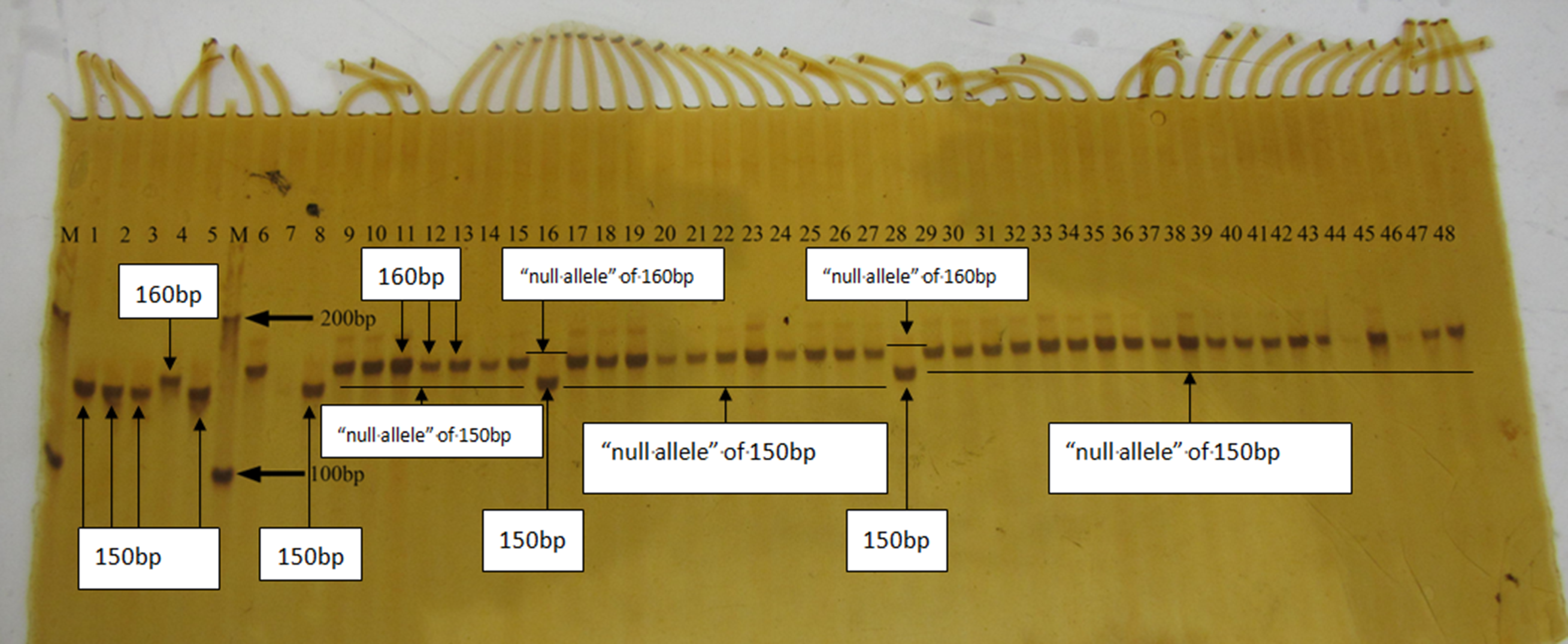

Supplement: Figure S1 — Profile amplified by RM6215 using total DNA of partial varieties (Variety A385 to A432) M: Marker, 1-48: rice varieties: 1, Yuedao 114; 2, Yuedao 115; 3, Yuedao 116; 4, Yuedao 117; 5, Yuedao 118; 6, Yuedao 119; 7, Yuedao 120; 8, Yuedao 121; 9, Jia 45; 10, Nannongjing 3786; 11, 24248; 12, Nannongjing 4004; 13, Nannongjing 4016; 14, Zijianwujing; 15, Ningjing 2hao; 16, Wuxiang 99-8; 17, Wuyunjing 8hao; 18, Nannongjing 002; 19, Nannongjing 004; 20, Huaidao 5hao; 21, Zhongzuo 93; 22, Yandao 9hao; 23, Lianjing 4hao; 24, Jindao 1007; 25, Huajing 5hao; 26, Huajing 6hao; 27, Yangfujing 7hao; 28, Yangfujing 8hao; 29, Zhendao 99; 30, Nanjing 42; 31, Lianjing 9823; 32, Huifeng 1hao; 33, Huifeng 2hao; 34, Yandao 8hao; 35, Wuyunjing 21hao; 36, Shashani; 37, Muzhan 4hao; 38, Mudanjiang 29; 39, Mudanjiang 28; 40, Mudanjiang 27; 41, Kenzhan 2hao; 42, Heijing 8hao; 43, Hejing 1hao; 44, Beidao 4hao; 45, Beidao 3hao; 46, Suijing 12hao; 47, Songjing 12; 48, Songjing 11. [file Image1.tif]

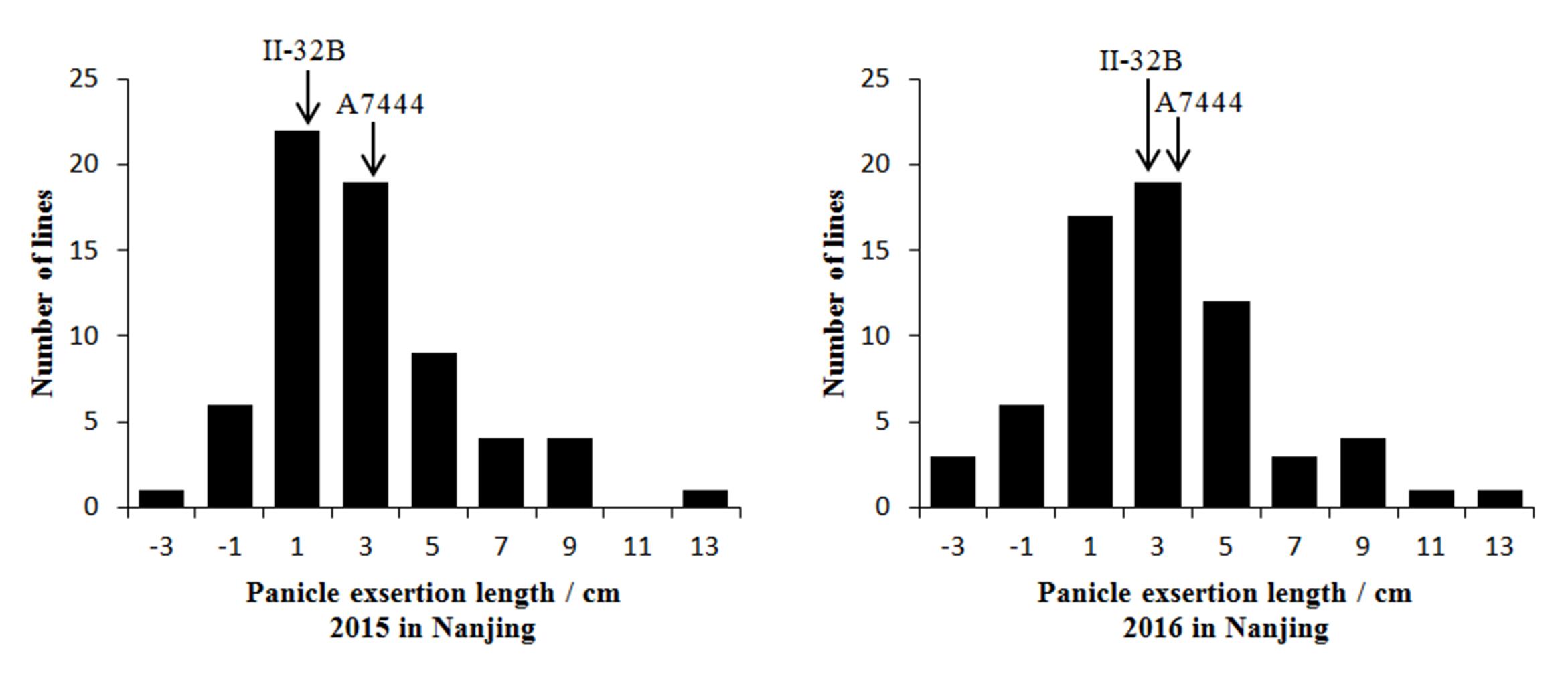

Supplement: Figure S2 — Frequency distribution of the PEL in the CSSL population under two growing environments. The parents of II-32B and A7444 were noted above the histograms. [file Image2.tif]

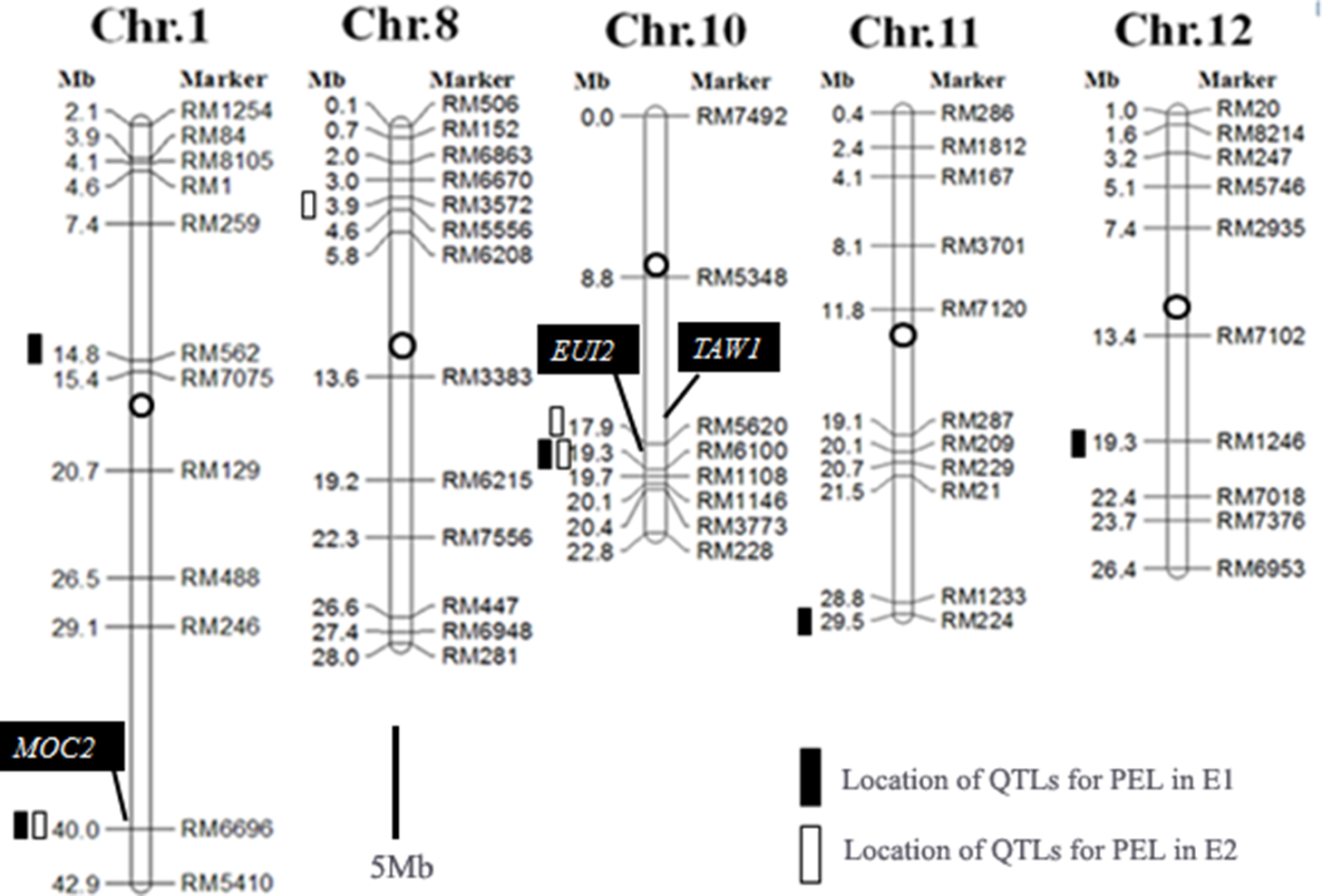

Supplement: Figure S3 — Chromosome locations of the QTLs for the PEL trait detected in the IIA-CSSL population and other reported genes of related traits. The black symbols stand for QTLs detected in E1; the white symbols stand for QTLs detected in E2. [file Image3.tif]

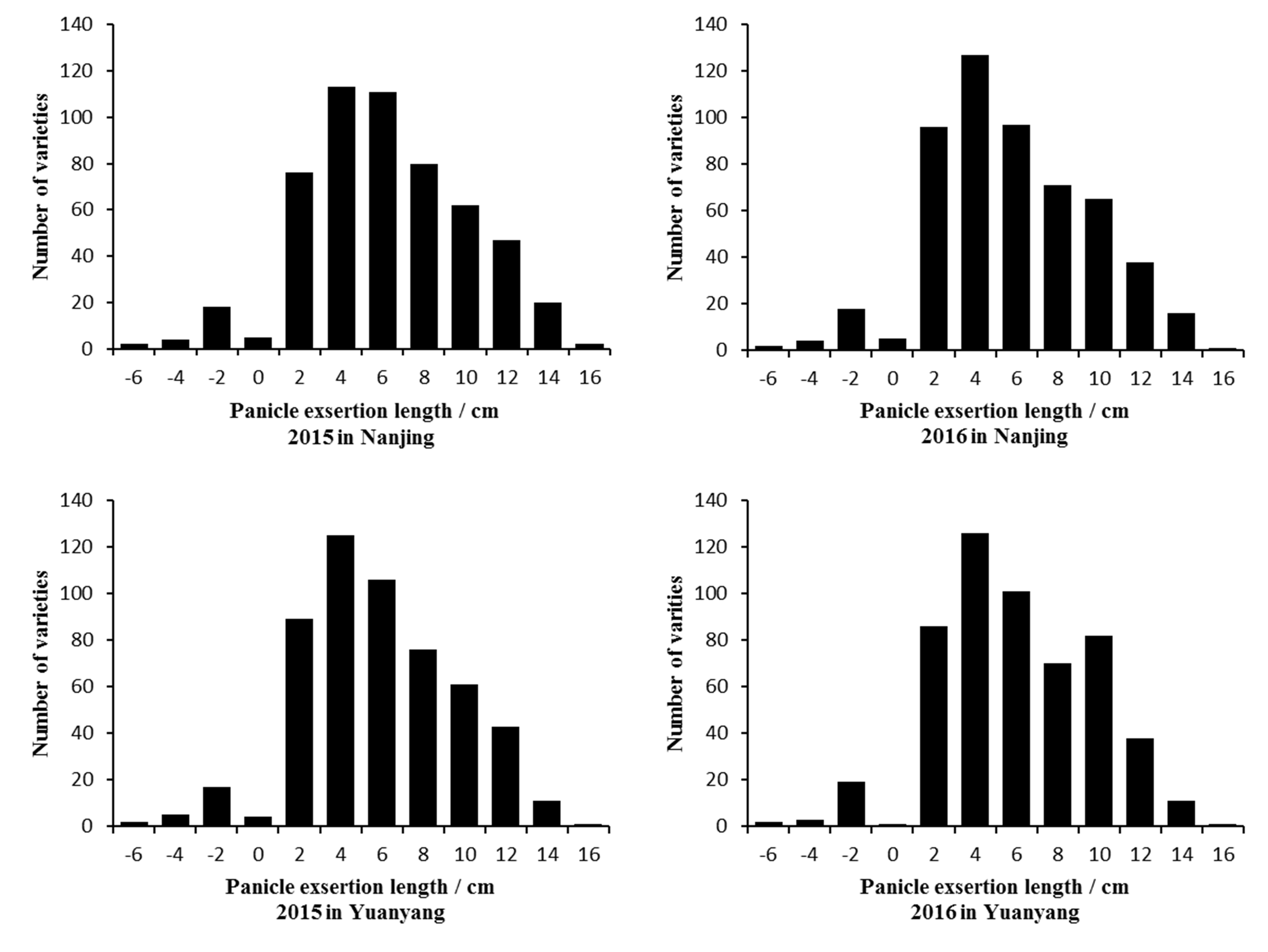

Supplement: Figure S4 — Frequency distribution of the PEL in the natural population under four growing environments. [file Image4.tif]

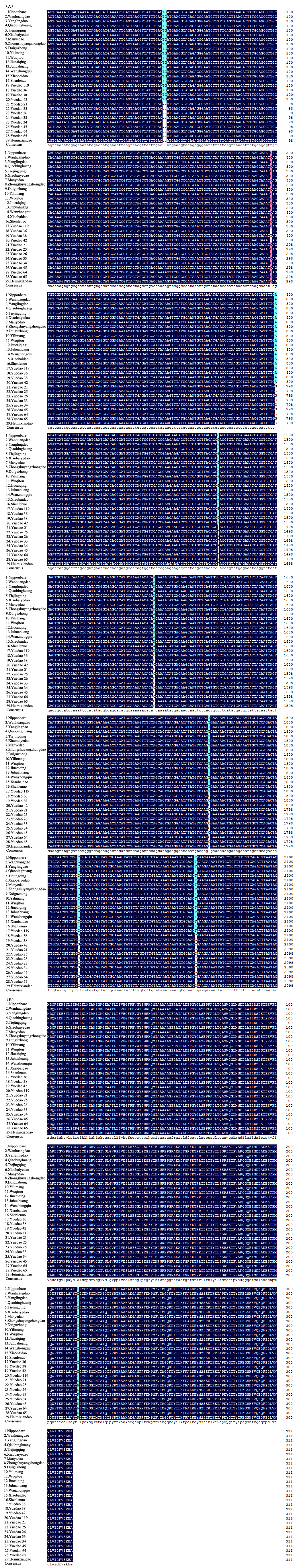

Supplement: Figure S5 — Gene sequence and deduced peptide sequence alignment of EUI2 between 25 accessions and Nipponbare. (A) Gene sequence. We show only the alignment results of the variance sequence; the alignment results of the no-difference sequence are not shown. (B) Deduced peptide sequence. [file Image5.tif]

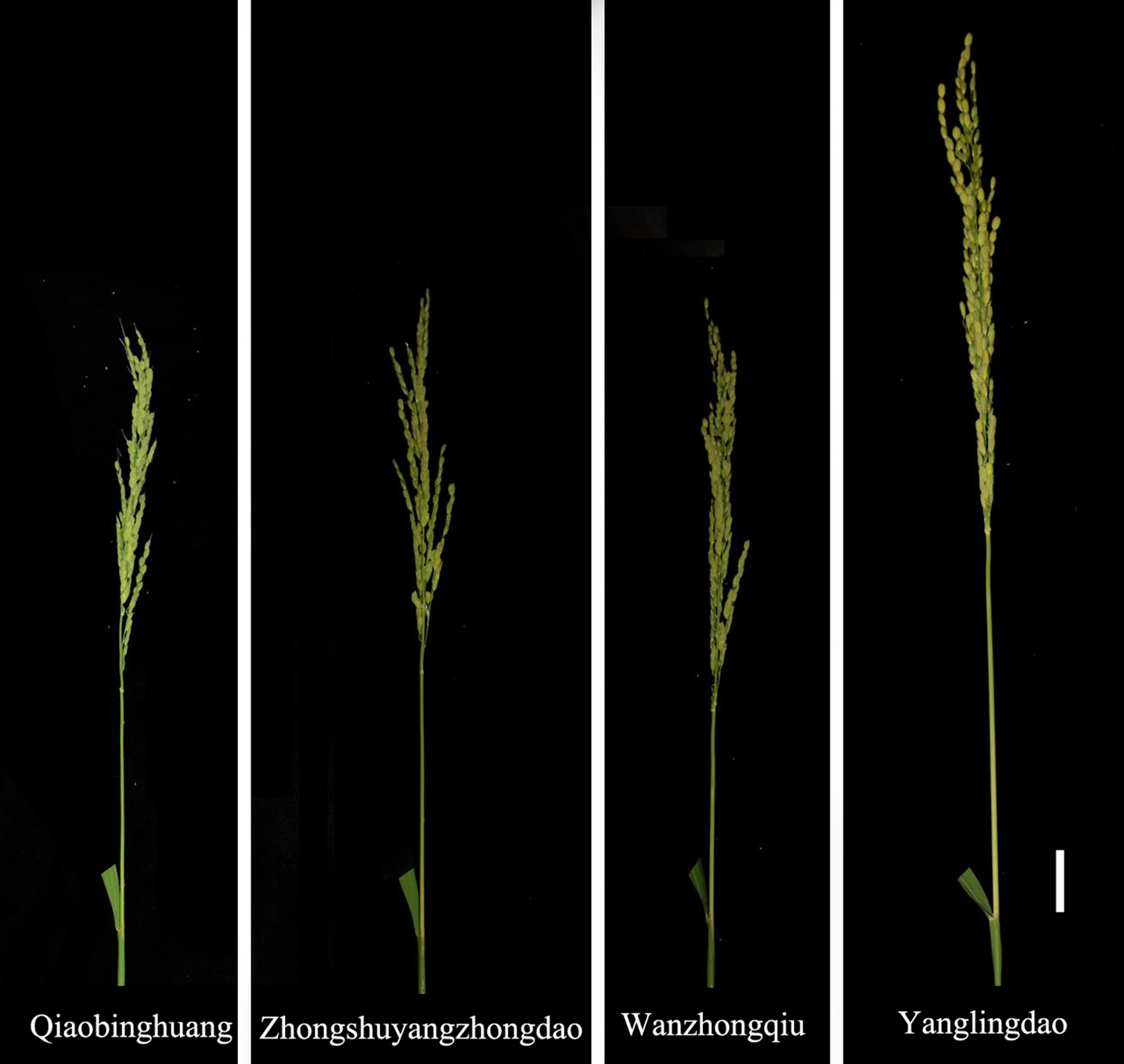

Supplement: Figure S6 — Morphology of the panicle for four superior parents. Scale bar, 3 cm. [file Image6.tif]
